# A Dual Repeat *Cis*-Element Determines Expression of *GERANYL DIPHOSPHATE SYNTHASE* for Monoterpene Production in *Phalaenopsis* Orchids

**DOI:** 10.3389/fpls.2018.00765

**Published:** 2018-06-05

**Authors:** Yu-Chen Chuang, Yi-Chu Hung, Chi-Yu Hsu, Chuan-Ming Yeh, Nobutaka Mitsuda, Masaru Ohme-Takagi, Wen-Chieh Tsai, Wen-Huei Chen, Hong-Hwa Chen

**Affiliations:** ^1^Department of Life Sciences, National Cheng Kung University, Tainan, Taiwan; ^2^Division of Strategic Research and Development, Graduate School of Science and Engineering, Saitama University, Saitama, Japan; ^3^Bioproduction Research Institute, National Institute of Advanced Industrial Science and Technology, Tsukuba, Japan; ^4^Institute of Tropical Plant Sciences, National Cheng Kung University, Tainan, Taiwan; ^5^Orchid Research and Development Center, National Cheng Kung University, Tainan, Taiwan

**Keywords:** *cis*-element, floral scent, monoterpene, orchid, *Phalaenopsis*, promoter, transcription factor, yeast-one hybrid screening

## Abstract

*Phalaenopsis bellina* is a scented orchid emitting large amount of monoterpenes. GERANYL DIPHOSPHATE SYNTHASE (PbGDPS) is the key enzyme for monoterpene biosynthesis, and shows concomitant expression with the emission of monoterpenes during flower development in *P. bellina*. Here, we identified a dual repeat *cis*-element in the *GDPS* promoter that is critical for monoterpene biosynthesis in *Phalaenopsis* orchids. A strong correlation between the dual repeat and the monoterpene production was revealed by examination of the *GDPS* promoter fragments over 12 *Phalaenopsis* species. Serial-deletion of the 2-kb *GDPS* promoter fragments demonstrated that the integrity of the dual repeat was crucial for its promoter activities. By screening the *Arabidopsis* transcription factors (TFs) cDNA library using yeast one-hybrid assay, AtbZIP18, a member of group I of bZIP TFs, was identified to be able to bind the dual repeat. We then identified *PbbZIP4* in the transcriptome of *P. bellina*, showing 83% identity in the DNA binding region with that of AtbZIP18, and the expression level of *PbbZIP4* was higher in the scented orchids. In addition, PbbZIP4 transactivated the *GDPS* promoter fragment containing the dual repeat in dual luciferase assay. Furthermore, transient ectopic expression of *PbbZIP4* induced a 10-fold production of monoterpenoids in the scentless orchid. In conclusion, these results indicate that the dual repeat is a real TF-bound *cis*-element significant for *GDPS* gene expression, and thus subsequent monoterpene biosynthesis in the scented *Phalaenopsis* orchids.

## Introduction

*Phalaenopsis* species are widespread in the tropical Asia regions and includes approximately 56 native species (Christenson, 2001). Numerous *Phalaenopsis* cultivars with diverse floral appearance are obtained via breeding and have become popular orchids due to their outstanding floral display and longevity (Hsiao et al., 2011a). In addition, some of the *Phalaenopsis* cultivars with pleasant fragrance improve their ornamental value in the floriculture market. However, breeding scented orchid cultivars under traditional breeding is difficult compared to other favorable traits (Yeh et al., 2014). The bottlenecks include long generation time (Hsiao et al., 2011a), cross-incompatibility due to the differences in genome size and chromosome size among species (Hsiao et al., 2011a,b; Yeh et al., 2014), and negative correlation between floral scent and other favorable traits (Hsiao et al., 2011b), which is also occurred in other modern floriculture varieties (Vainstein et al., 2001; Dudareva and Negre, 2005). In such circumstances, alternative approaches to facilitate scented orchid breeding are needed.

The majorities of *Phalaenopsis* orchids are scentless but some do emit scent volatile organic compounds (VOCs) (Kaiser, 1993). These scented species have been extensively used as breeding parents for production of scent cultivars, such as *P. amboinensis*, *P. bellina*, *P. javanica*, *P. lueddemanniana*, *P. schilleriana*, *P. stuartiana*, *P. venosa*, and *P. violace* (Hsiao et al., 2011b; Yeh et al., 2014). Both *P. bellina* and *P. violacea* are two very close species popular in breeding scented phenotype and emits similar but distinct floral VOCs. *P. bellina* emits mainly monoterpenoids, including citronellol, geraniol, linalool, myrcene, nerol, and ocimene (Hsiao et al., 2006, 2011b), while *P. violacea* emits monoterpenoids accompanied with a phenylpropanoid, cinnamyl alcohol (Kaiser, 1993). The VOCs of *P. schilleriana* contain monoterpenoids as well, including citronellol, nerol and neryl acetate (Awano et al., 1997).

Monoterpenoids, the most abundant constituent in volatile terpenoids (Knudsen and Gershenzon, 2006; Nagegowda et al., 2010), are involved in specialized interactions with other organisms and surrounding environment (Tholl, 2015), for example, to function as attractants for pollinators (Byers et al., 2014), as antibacterial and antifungal compounds (Hammer et al., 2003; Yamasaki et al., 2007; Marei et al., 2012; Mirzaei-Najafgholi et al., 2017), and for defense against herbivores (Paré and Tumlinson, 1999; Mumm et al., 2008; Huang et al., 2018). Apart from their roles in nature, monoterpenoids are widely used in flavor, cosmetics, and perfumery industries due to their unique and pleasant fragrance characteristics (Schwab et al., 2008). In addition, they are exploited as health-promoting compounds and have potential to be applied in cancer therapeutics because of their anti-cancer activities (Gould, 1997; Crowell, 1999; Carnesecchi et al., 2001; Jun et al., 2006; Murthy et al., 2012; Cho et al., 2016; Nakayama et al., 2017).

The precursors of monoterpenoids, IDP and its isomer, DMADP, are produced from the methylerythritol phosphate (MEP) pathway in the plastid. The short-chain prenyltransferases, GDPS, is responsible for the head-to-tail condensation of IDP and DMADP to generate the direct substrate GDP for monoterpene synthases (Dudareva et al., 2004). In *Phalaenopsis* orchids, PbGDPS is characterized as the key enzyme to provide precursors for monoterpene biosynthesis in *P. bellina* (Hsiao et al., 2008). Interestingly, recombinant PbGDPS possesses dual prenyltransferase activities for the production of both GDP and farnesyl diphosphate (FDP), the precursor for monoterpeneoids, and sesquiterpenoids, respectively (Hsiao et al., 2008). Expression of *PbGDPS* is concomitant with the emission of monoterpenoids during flower developments, peaked on day 5 post anthesis (D + 5) (Hsiao et al., 2008).

To date, promoters of the genes in the terpenoid biosynthesis pathway have been investigated and functionally assayed in several species. The vascular-specific expression of a hydroxymethylbutenyl 4-diphosphate synthase gene (*HDS*) promoter is identified from the MEP pathway in *Catharanthus roseus* (Ginis et al., 2012). The leaf-specific expression of a geranylgeranyl diphosphate synthase gene (*SmGGPPs*) promoter is analyzed in *Salvia miltiorrhiza* (Hua et al., 2012). The vegetative organ-specific expression of a mevalonate diphosphate decarboxylase (*GbMVD*) from MVA pathway is examined in *Ginkgo biloba* (Liao et al., 2016). For defense responses, the promoters of four sesquiterpene synthase genes for β-caryophyllene (*CPS*), epi-cedrol (*ECS*), β-farnesene (*FS*), and amorpha-4,11-diene synthase (*ADS*), and one monoterpene synthase gene (*LIS*) are studied in *Artemisia annua* (Wang et al., 2013, 2014). In addition, a number of *cis*-acting elements on the promoters of terpenoid biosynthesis pathway genes have been identified. A W-box palindrome is identified in the promoter of a cotton sesquiterpene synthase gene (*CAD1-A*) for GaWRKY1 binding (Xu et al., 2004). The E-box on the promoters of two *Arabidopsis* sesquiterpene synthase genes *TPS21* and *TPS11* is recognized by MYC2 involved in gibberellin and jasmonate induction (Hong et al., 2012). The NAC binding sites are found in the promoter of a monoterpene synthase gene (*AaTPS1*) in the scented kiwifruit *Actinidia arguta* for AaNAC2, 3 and 4 binding, which are mutated in the scentless *A. chinensis* (Nieuwenhuizen et al., 2015). The GCC-box is identified in the promoter of the terpene synthase gene (*TPS10*) for ZmEREB58 binding in *Zea mays* (Li et al., 2015), and in the promoter of *CitTPS16* for CitERF71 in *Citrus* fruit (Li et al., 2017).

In this study, we reported that a dual repeat in the upstream promoter fragments of *GDPS* is essential for its transcriptional activation in *Phalaenopsis* orchids. The full dual repeat was present only in the *Phalaenopsis* orchids emitting monoterpenes, and its integrity showed strong association with the transactivation of a bZIP TF, bZIP4. As this dual repeat was closely related to the monoterpene production in *Phalaenopsis* orchids, it could be developed as a promising molecular marker for early detection of monoterpene phenotype in the offspring and thus facilitate scented orchid breeding in future.

## Materials and Methods

### Plant Materials and Growth Condition

Ten native and two cultivar hybrids were used in the study (Supplementary Figure 1), including *P. amboinensis* var. *yellow* (abbreviated as *P. amboinensis*), *P. aphrodite* subsp. *formosana* (abbreviated as *P. aphrodite*), *P. bellina*, *P. cornu-cervi* var. *red* (abbreviated as *P. cornu-cervi*), *P. equestris* ‘RO-5’, *P. equestris* ‘WY-7’, *P. javanica*, *P. lueddemanniana*, *P. mannii*, *P. schilleriana*, *P.* I-Hsin Venus ‘KHM2212’ (abbreviated as *P.* I-Hsin Venus), and *P.* Meidarland Bellina Age ‘LM128’ (abbreviated as *P.* Meidarland Bellina Age). These individual plants were collected from various orchid nurseries across Taiwan (details in Supplementary Table 1). Both *P.* I-Hsin Venus and *P.* Meidarland Bellina Age are commercial scented cultivars with monoterpenes as their major scent compounds (Chuang et al., 2017), whose ancestries contain multiple scent species as the following: *P.* I-Hsin Venus - *P. amboinensis* (25%), *P. equestris* (25%), *P. venosa* (18.75%), *P. violacea* (15.63%), *P. lueddemanniana* (3.13%); *P.* Meidarland Bellina Age - *P. bellina* (50%), *P. violacea* (24.22%), *P. venosa* (15.63%), *P. amboinensis* (3.91%), *P. lueddemanniana* (3.13%) via ORCHIDEYA.CA^1^. All the plant materials were grown in the greenhouse at National Cheng Kung University (NCKU) under natural light and surrounding temperature from 27 to 30°C in spring and summer with 75–85% humidity.

### Gas Chromatographic Analysis of Floral Volatiles

Analysis of the floral VOCs of 12 *Phalaenopsis* orchids was carried out according to the previous studies (Hsiao et al., 2006; Chuang et al., 2017). The VOCs were collected during the most emitted scent period (from 10:00 to 16:00) by using solid phase extraction system (DSC-Si and DCS-18, Supelco, United States) as described (Chuang et al., 2017), and the compounds were then identified by using gas chromatography/high-resolution mass spectrometry (GC/HRMS) at the NCKU Instrument Center (Hsiao et al., 2006). To assess the amounts of each compound, 1 μg of ethyl myristate was recruited as the internal standard (Fluka, Honeywell, United States).

### Detection of *GDPS* Gene Sequence, Upstream Regulatory Fragment and the Dual Repeat Region in 12 Orchid Genomes

To detect the *GDPS* gene and its upstream regulatory fragment, the genomic DNA of 12 *Phalaenopsis* orchids were extracted by using Plant Genomic DNA Purification Kit (Bio-GPD50, Biokit, Taiwan). Standard PCRs were applied to amplify the N-terminal region of *GDPS* (∼400-bp) with the primer designed based on *PbGDPS* genomic sequence (all the primers used here and thereafter were listed in Supplementary Table 2) since *PbGDPS* is an intronless gene (Hsiao et al., 2008). The 1-kb upstream promoter fragments of *GDPS* were also isolated from the 12 *Phalaenopsis* orchids using the designed primers based on the genomic DNA of *P. bellina* (Chuang et al., 2017). The dual repeat region was then amplified and cloned with ZeroBack Fast Ligation Kit (TIANGEN, China). Six to eight colonies were selected randomly for sequencing. The presence of the *cis*-elements in the dual repeats was predicted using PlantPAN (Chow et al., 2015), with 100% similar score accepted as the predicted results.

### Quantitative Real-Time RT-PCR

Total RNA was extracted from the flowers on D + 5 stage of the 12 *Phalaenopsis* orchids following the protocol of Plant Total RNA Miniprep Purification Kit (TR02, GeneMark, Taiwan). Reverse transcription to cDNA involved use of SuperScript III (Thermo Fisher Scientific, United States). Primers were designed by using Primer Express 3.0 (Thermo Fisher Scientific, United States). Quantitative RT-PCR (qRT-PCR) was performed by using the ABI StepOne Plus Quantitative real-time PCR instrument and SYBR Green kit (Applied Biosystems, United States) as described (Hsu et al., 2015). All expression results were normalized to the reference gene, *PbActin1* (Chuang et al., unpublished). Mean and standard error were calculated from triplicate repeats.

### Plasmid Construction

The 2-kb promoter fragment upstream from the translation start site of *PbGDPS* (denoted as *PbG*p-2010) and its nine serial deletion fragments (*PbG*p-1076, *PbG*p-859, *PbG*p-784, *PbG*p-710, *PbG*p-584, *PbG*p-410, *PbG*p-354, *PbG*p-297, *PbG*p-216) (**Figure 4A**) were amplified from the genomic DNA of *P. bellina*. Further serial-deletion fragments (*PbG*p-836, *PbG*p-822, *PbG*p-760, *PbG*p-747, *PbG*p-729) (**Figure 4B**) were amplified for detail analysis of the region between -836 bp to -710 bp. Specific primers with the restriction endonuclease sites of *Bam*H I and *Nco* I were designed to amplify these truncated fragments. The amplified fragments were double-digested with the restriction enzyme *Bam*H I and *Nco* I, and cloned into the corresponding enzyme digestion sites of pJD301(f) to drive the firefly (*Photinus pyralis*) luciferase gene (Hsu et al., 2014). All constructs were verified by DNA sequencing. The promoter-*LUC* constructs were schematically presented in **Figure 4**.

### Transactivation Assay of *PbGDPS* Promoter Fragments *in Planta*

The promoter-*LUC* constructs were bombarded into the floral tissues of *P.* I-Hsin Venus with an internal control plasmid, pJD301(R), containing the *Renilla* luciferase gene driven by cauliflower mosaic virus (CaMV) 35S promoter. For normalization, the luciferase activity of the reporter construct was divided by that of the internal control. The involvement of internal control reduced experimental variability resulted from differential bombardment efficiency and transformation efficiency among various experimental groups. The amount of the reporter plasmid and the internal control was 10 and 0.1 μg, respectively. At least six individual flowers of *P.* I-Hsin Venus were employed for replicates. Luciferase activity of each sample was measured (Hsu et al., 2014). For statistics analysis between two groups, pairwise comparisons were performed by using Tukey’s honestly significant difference test at α = 0.05.

### Yeast One-Hybrid (Y1H) Library Screening

Systematic screening of Y1H TF library composed of approximately 1,350 *Arabidopsis* TFs (Mitsuda et al., 2010) was performed in yeast strain YM4271 to identify the TFs that are able to bind the dual repeat. For construction of bait plasmid, the dual repeat was amplified from the genomic DNA of *P. bellina* by using appropriate primers with the restriction endonuclease site of *Xma* I and *Xba* I, cloned into pHisi2, and integrated it into the yeast genome. The Y1H assay was performed as previously described (Mitsuda et al., 2010). In the screenings of *Arabidopsis* TF library, the degree of positive interaction between a prey TF and a bait sequences is scored between 0 and 3 in each screening according to the yeast growth status under selective media so that each TF has its own “sticky score” as the sum of this score. The sticky score of AtbZIP29 and AtbZIP30 was 50 and 24, respectively, among 247 TFs isolated so far in 105 screenings including this study and therefore were considered as sticky TFs in this Y1H system.

### Identification of bZIP Group I TFs in *Phalaenopsis* Transcriptome

The bZIP group I TFs was identified in the *P. bellina* transcriptomic data of four floral development stages at anthesis day (Dd), D + 3, D + 5, and D + 7, which corresponds to the four periods of the floral monoterpene emission pattern, including onset, increase, peak and decline (Chuang et al., unpublished), by using those in *Arabidopsis* as queries with *E*-value cutoff of 1e^-5^ (Jakoby et al., 2002; Pyo et al., 2006). The expression level of each individual TF gene was presented by fragments per kilobase of transcript per million mapped reads (FPKM). FPKM values of the *P. bellina* transcriptome were transformed by log 3.22 to achieve equivalent expression levels of reference genes as those in the transcriptomic data of *P. aphrodite* (Su et al., 2013b), including *Actin4* (Chen et al., 2005; Hsieh et al., 2013; Pan et al., 2014; Hsu et al., 2015), *Actin9* (Hsiao et al., 2008; Pan et al., 2011, 2014; Hsu et al., 2015), and *Ubiquitin10* (Lu et al., 2007; Hsiao et al., 2008) (Chuang et al., unpublished). A multiple sequence alignment of bZIP domains was generated by using Clustal Omega^2^ and displayed by using BOXSHADE^3^. The phylogenetic tree was built with the neighbor-joining method with 1000 bootstrap trials by using MEGA6 (Tamura et al., 2013).

### Examination of the Transactivation of PbbZIP4 and PbbZIP26

Promoter fragments of *PbGDPS* and *PaGDPSpA*/*PaGDPSpB* were isolated from *P. bellina* and *P. aphrodite* genomic DNA, respectively (Chuang et al., unpublished). After sequence confirmation, these promoter fragments were cloned into pJD301(f) to drive the firefly luciferase gene. Coding sequences for both *PbbZIP4* and *PbbZIP26* were amplified with gene-specific primers from full-bloom flowers of *P. bellina* and cloned into pBI221 and under the control of CaMV 35S promoter. Three separate plasmids, including pBI221 with *PbbZIP4/26*, pJD301(f) with promoter fragments, and internal control pJD301(R), were co-bombarded into *P. aphrodite* floral tissues at a ratio of 1.5: 1.5: 0.15 (total 3.15 μg) as described previously (Hsu et al., 2014). The luciferase activity of each sample was measured after 20 h post bombardment. The relative fold change in activity was calculated by the comparison to the control assay with *GUS* in pBI221 for at least triplicate biological repeats. For statistics analysis between two groups, pairwise comparisons were performed by using Tukey’s honestly significant difference test at α = 0.05.

### Transient Ectopic Expression of *PbbZIP4* in the Scentless Orchid

The coding sequence of *PbbZIP4* was isolated from the pBI221 plasmid containing *PbbZIP4* described above, and cloned into the p1304NhXb vector under a duplicated CaMV 35S promoter. The *Agrobacterium tumefaciens* EHA105 cells taking the resulting plasmids were infiltrated into the perianths of *P. aphrodite* at the Dd stages (Hsu et al., 2015). The empty plasmid vector containing *GUS* served as a negative control. Three individual flowers of *P. aphrodite* were employed for replicates. The volatiles of infiltrated flowers were collected on day 4 post infiltration for 6 hr (from 10:00 to 16:00) and the compounds were identified by GC/HRMS as described above. Total RNA was isolated from the infiltrated tissues following the protocol of RNeasy Plant Mini Kit (QIAGEN, Germany). Reverse transcription to cDNA and quantitative RT-PCR was performed as described above.

## Results

### Isolation of a Dual Repeat in the *GDPS* Upstream Promoter

**FIGURE 1:**
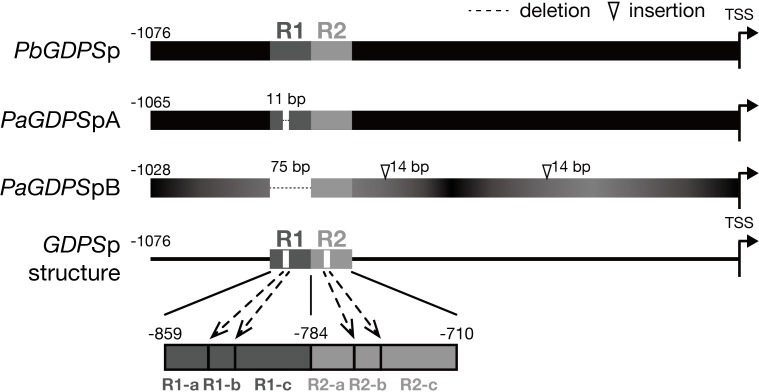
Promoter structure of *GDPS*. Promoter structure of *GDPS* was revealed by the comparison of three sequences of *PbGDPS*p, *PaGDPS*pA, and *PaGDPS*pB. Two repeats located from –859 to –710 of *PbGDPS*p were named as R1 and R2. The repeat was further dissected into three subunits based on 11-bp deletion located in the center of R1. This deletion was referred as R1-b, and the sequences prior to and behind R1-b was R1-a and R1-c, respectively. The corresponding dissection in R2 was R2-a, R2-b, and R2-c. TSS indicates the translation start site (ATG). Black color gradient in *PaGDPS*pB indicated its numerous substitutions compared to *PbGDPS*p.

Previously, two individual 1-kb fragments of *GDPS* promoters were isolated from the scentless *P. aphrodite*, namely *PaGDPS*pA and *PaGDPS*pB (Chuang et al., unpublished). Compared to the *GDPS* promoter from the scented *P. bellina* (*PbGDPS*p), two *GDPS* promoters identified from *P. aphrodite*, *PaGDPS*pA and *PaGDPS*pB contained an 11-bp deletion and a 75-bp deletion, respectively (**Figure 1**). *PaGDPS*pB also had two 14-bp insertions in addition to numerous nucleotide substitutions. By performing the luciferase promoter assays *in planta*, *PaGDPS*pA showed the similar promoter activity as *PbGDPS*p either in scented or scentless flowers, while *PaGDPS*pB revealed very low promoter activity even in the scented *P. bellina* floral tissues. These results indicated that the lack of the 75-bp region in the *PaGDPS*pB is detrimental for its activity (Chuang et al., unpublished). Further sequence analysis of the *PbGDPS* promoter showed that a second 75-bp repeat is present downstream from the original 75-bp repeat and formed a dual repeat consisted of the two 75-bp units. The first and second 75-bp units were then denoted as ‘R1’, and ‘R2’, respectively, located from -859 to -710 nt upstream from the ATG (**Figure 1**).

The *PaGDPS*pB lacked the entire R1 unit, and *PaGDPS*pA harbored a 11-bp deletion in the center of R1, which was defined as R1-b subunit (**Figure 1**). The region (25-bp) prior to the R1-b was denoted as R1-a, and those (39-bp) behind was R1-c, and the corresponding divisions in R2 were denoted as R2-a, R2-b and R2-c (**Figure 1**). The dual repeat structure was schematically represented in **Figure 1**, and the sequence of the dual repeat is in Supplementary Figure 2. The difference between the *GDPS* promoters of the scent *P. bellina* and the scentless *P. aphrodite* resided in the dual repeat, and this is well correlated with the monoterpene phenotype.

### Concomitance of the Integrity of the Dual Repeat With the Monoterpene Production

According to the promoter analysis results of *PaGDPS* and *PbGDPS* from scented and scentless *Phalaenopsis* orchids, we hypothesized that the dual repeat is associated with the monoterpene production. To confirm this, another 10 frequently used breeding parents of *Phalaenopsis* orchids (Supplementary Figure 1) were recruited and assessed for the correlation analysis between the dual repeat and the monoterpene production.

**FIGURE 2:**
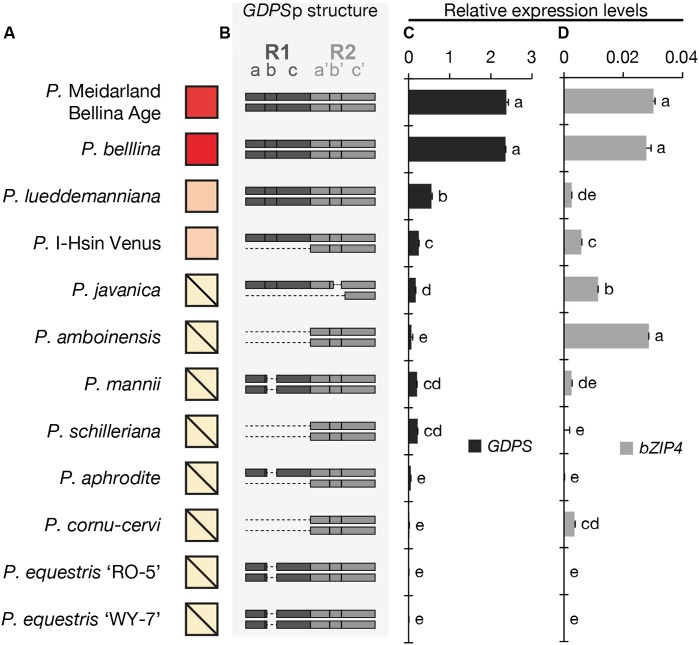
The analysis of the floral volatiles, dual-repeat and gene expression in 12 *Phalaenopsis* orchids. **(A)** The relative amounts of floral volatiles were represented by a red color gradient. The block with a diagonal line indicates that no monoterpenoid is identified in this orchid. **(B)** The dual repeat structure of *GDPS* promoter. **(C,D)** The expression levels of *GDPS* **(C)**, and *bZIP4* **(D)**. Expression level was normalized to that of *PbActin1* by real-time RT-PCR. Data are mean ± SE from triplicate measurements. Statistic tests were performed by using Tukey’s honestly significant difference test at α = 0.05.

We first examined the floral scent profile (Supplementary Table 3) and found that four orchids emitted monoterpenoids, including *P.* Meidarland Bellina Age, *P. bellina*, *P.* I-Hsin Venus, and *P. lueddemanniana*. In contrast, the major VOCs of *P. javanica* and *P. amboinensis* were sesquiterpenoids and benzenoids, and that of *P. mannii* was phenylpropanoids and fatty acid derivatives (Supplementary Table 3). *P. schilleriana* emitted trace amounts of benzenoids. *P. aphrodite*, *P. cornu-cervi*, *P. equestris* ‘RO-5’, and *P. equestris* ‘WY-7’ were considered as “scentless” since no scent compounds were detected (Supplementary Table 3). For brief, the relative amounts of monoterpenoids emitted from these *Phalaenopsis* orchids were symbolized in **Figure 2A**.

**FIGURE 3:**
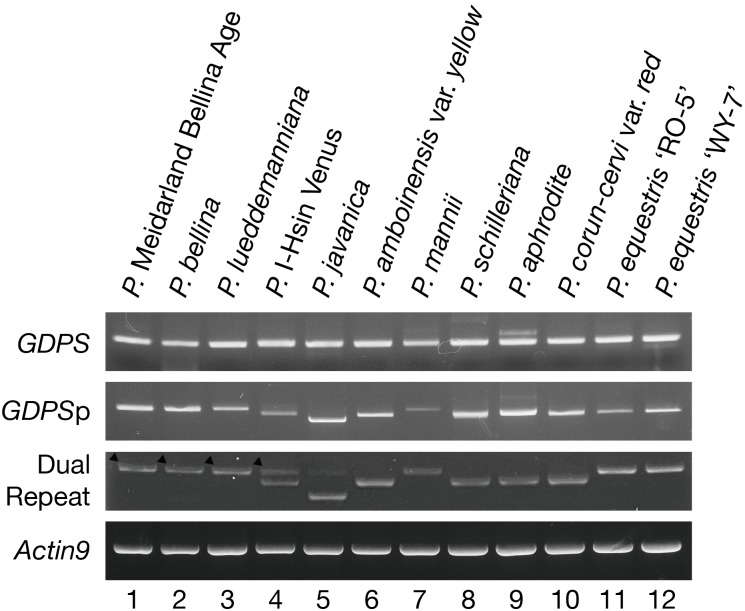
PCR Amplification of the *GDPS* gene, its 1-kb promoter fragment and the dual repeat in the genomic DNA of 12 *Phalaenopsis* orchids. Actin was used as a control. Amplification of 1-kb promoter and dual repeat showed polymorphisms.

The presence of the *GDPS* gene and its promoter sequence in the 12 *Phalaenopsis* orchids were then analyzed (**Figure 3**). Intriguingly, the *GDPS* gene was present in all of these orchids regardless of being scent or scentless phenotype (**Figure 3**). It is plausible that the defects are resided in the promoter region (*GDPS*p, **Figure 3**). We then amplified the dual repeat on *GDPS*p and a polymorphism of the dual repeat fragment length was detected among the 12 *Phalaenopsis* orchids (**Figure 3**). The four scented orchids with monoterpene production contain the complete dual repeat (**Figure 3**, the black arrowheads). In contrast, the amplified dual repeat fragments of the other orchids were reduced to various extents with various deletions in the dual repeat region. These 12 fragments were cloned and sequenced. Deletions in the dual repeats were detected between nucleotides 11 and 110, which appear to cause defects in *GDPS* promoter activities in the orchids without monoterpene production (**Figures 2A,B**). Strikingly, most defects occurred in the R1 region (**Figure 2B**).

We then examined the expression levels of *GDPS* for 12 *Phalaenopsis* orchids (**Figure 2C**). The four orchids emitting monoterpenoids especially both *P.* Meidarland Bellina Age and *P. bellina* showed higher *GDPS* expression levels than the others (**Figure 2C**). Taken together, we concluded that the integrity of the dual repeat in the *GDPS* promoter is strongly correlated with its elevated expression and thus the monoterpene production.

### The Dual Repeat Is Crucial for *GDPS* Promoter Activity

To investigate the role of the dual repeat in the promoter activity of *GDPS*, the ∼2-kb promoter fragment (denoted *PbG*p-2010) upstream from the start site of *PbGDPS* was isolated and subjected to serial deletions. The activity of *PbG*p-2010 and the nine truncated promoter fragments were evaluated in *P.* I-Hsin Venus flowers via particle bombardment for dual luciferase assays. It was legitimate that we should examine *PbGDPS* promoter activity in the original species *P. bellina.* However, the supply of *P. bellina* flowers fell short of demand for experiments as *P. bellina* commonly produces only one flower per 20 days. Instead, *P.* I-Hsin Venus, the offspring of *P. bellina* emitting similar scents, was micropropagated to large quantities with the identical genetic background and would help to reduce variation.

**FIGURE 4:**
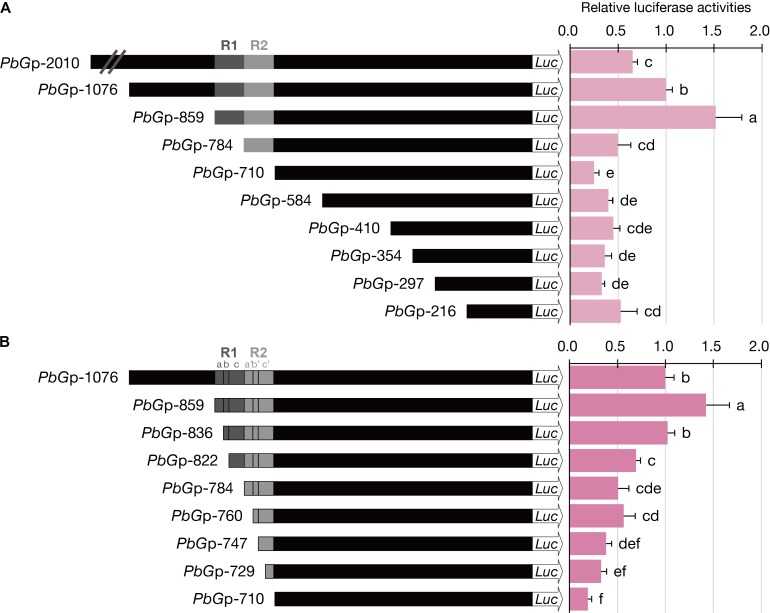
Luciferase activities of dissected *PbGDPS* promoters in *P.* I-Hsin Venus by particle bombardment. **(A)** Serial deletion of *PbGDPS* promoter to analyze the putative *cis*-element. **(B)** Identification of the subunit of the dual repeat responsible for promoter driving abilities. Activation level was given by the ratio of Luc/RLuc and the level of *PbGp*-1076 was set as 1. Experiments are based on at least sextuplicate biological replicate. Statistic tests were performed by using Tukey’s honestly significant difference test at α = 0.05.

The highest luciferase activity was observed for *PbG*p-859, which showed approximately threefold increase as compared to that *PbG*p-784, and fivefold increase as compared to that of *PbG*p-710 (**Figure 4A**). Thus, the *cis*-element responsible for high promoter activity was between nucleotide (nt) -859 and nt -710 (150-bp), in which the dual repeat located. Further dissection of *PbG*p-710 to generate *PbG*p-584, *PbG*p-410, *PbG*p-354, *PbG*p-297, and *PbG*p-216 showed that no extra enhancers were present at the downstream of the dual repeat (**Figure 4A**). Compared with *PbG*p-859, the promoter activity of *PbG*p-2010 and P*bG*p-1076 was much decreased (**Figure 4A**), suggesting the presence of repressor elements at the upstream region of *PbG*p-859. These results verified that the dual repeat plays a crucial role for *PbGDPS* promoter activity.

To further define the *cis*-element in the 150-bp dual repeat region for transcriptional regulation, a more detailed analysis was performed based on the subunit division, namely R1a, R1b, R1c, R2a, R2b, and R2c. These subunits were serial deleted to generate a series of truncated promoter constructs. The luciferase activities were reduced further with gradual deletions of the subunit, indicating the essential nature of the complete dual repeat for full promoter activity of *PbGDPS* (**Figure 4B**).

### Yeast One-Hybrid Screening of the Transcription Factor Bound Onto the Dual Repeat

To identify the candidate upstream *trans*-activators, Y1H screening was performed using *P. bellina* floral cDNA as preys. However, the dual repeat bait produced extensive background growth of yeast, and it could not be eliminated even under the addition of inhibitor 3-Amino-1,2,4-triazole (3-AT). Alternatively, Y1H screening against prey library composed of approximately 1,350 TFs of *Arabidopsis thaliana* was performed (Mitsuda et al., 2010). The dual repeat was amplified from *P. bellina* genomic DNA and fused to a minimal promoter of *HISTIDINE SYNTHASE3* (Supplementary Figure 3A). In this approach, the leaky expression of the reporter gene could be overcome by the addition of 4 mM 3-AT (Supplementary Figure 3B). Total four positive TFs were obtained from this screening (**Table 1**) and three of them encoded bZIP family proteins including AtbZIP18, AtbZIP29, and AtbZIP30. The other one candidate AtAGL81, a MADS-box TF, failed to be isolated from *P. bellina* floral transcriptome.

**Table 1:** Transcription factors binding to the dual repeat by screening of yeast one-hybrid libraries.

Screening of Y1H library	Isolated TF		TF family	Interaction strength	Homology gene in *P. bellina* floral transcriptome
	Locus	Name			
*Arabidopsis*	AT2G21230	AtbZIP30^a^	bZIP	Weak	—
	AT2G40620	AtbZIP18	bZIP	Weak	Yes
	AT4G38900	AtbZIP29^a^	bZIP	Strong	—
	AT5G39750	AGL81	MADS	Strong	No

*^*a*^AtbZIP29 and AtbZIP30 were excluded for following analysis due to their sticky feature in the system.*

**FIGURE 5:**
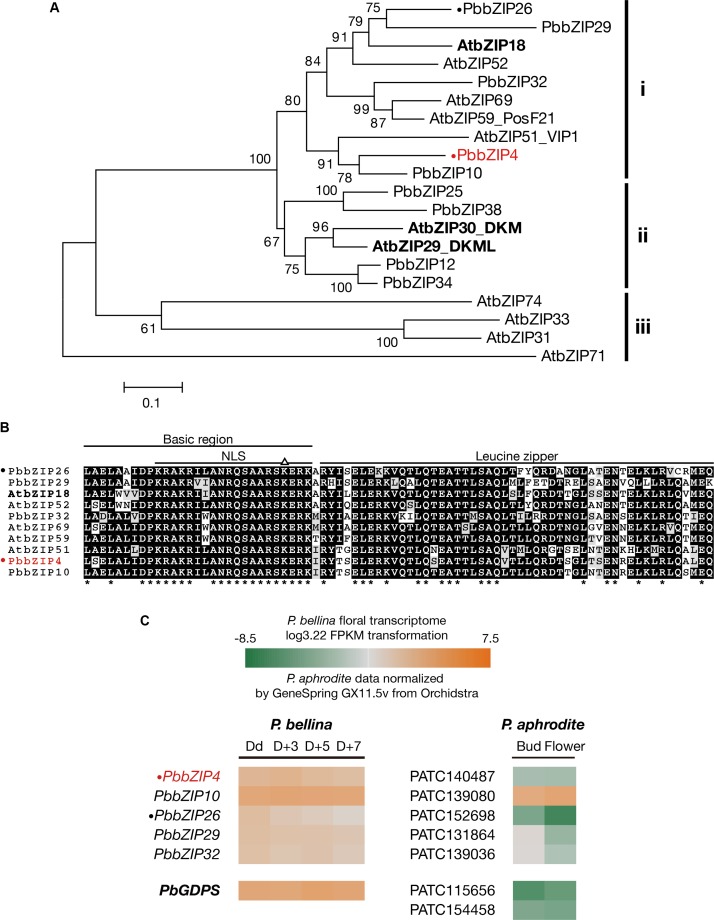
Analysis of bZIP group I TFs in *P. bellina* floral transcriptome. **(A)** Phylogenetic tree inferred from the amino sequences of the nine bZIP group I TFs with the ones in *Arabidopsis*. The three subgroups were divided based on their phylogenetic relationship. Three AtbZIPs isolated by Y1H screening were seen in bold. **(B)** Amino acid sequence alignment of subgroup i proteins in **(A)**. Asterisks (^∗^) below the alignment indicate the consensus residues in all sequences. The triangle (∆) above the alignment indicates the lysine replacement of the conserved arginine residue in the basic region of group I of bZIP family. **(C)** Comparison of the expression levels of five bZIP TFs between two *Phalaenopsis* transcriptomes. Gene expression levels in *P. bellina* transcriptomes were transformed by log3.22 FPKM in four floral developmental stages, including Dd, D + 3, D + 5, and D + 7 (Chuang et al., unpublished), while the levels of two floral stages of flower bud and flower in *P. aphrodite* transcriptomes were analyzed via microarray experiments stored in Orchidstra (Su et al., 2013a). Expression levels of putative genes are represented by a color gradient from orange to gray to green. Two PbbZIPs for transient assays, PbbZIP4 and PbbZIP26, are labeled with a dot (•). PbbZIP4 was especially shown in red. The expression levels of *PbGDPS* (in bold) was also included for comparison.

The three bZIP TFs belong to the group I of bZIP family composed of a conserved bZIP domain for DNA binding and a leucine zipper motif for dimerization (Jakoby et al., 2002). The *P. bellina* floral transcriptomic data (Chuang et al., unpublished) showed nine proteins belong to group I, and can be divided into three subgroups (i, ii, and iii) according to their phylogenetic relationship with the *Arabidopsis* ones (**Figure 5A**). We selected subgroup i including AtbZIP18 for further analysis since AtbZIP29 and AtbZIP30 (subgroup ii) were repeatedly isolated in other unrelated screenings and therefore considered as sticky factors in the Y1H system. No PbbZIPs were classified into subgroup iii (**Figure 5A**). Multiple alignments showed that five PbbZIPs namely PbbZIP4, PbbZIP10, PbbZIP26, PbbZIP29, and PbbZIP32 in the subgroup i shared 83–86% identity with AtbZIP18 in the basic region (**Figure 5B**).

Previously, we found that the promoter activity of *GDPS* was much higher in the scented *P. bellina* than in the scentless *P. aphrodite*. In addition, *PaGDPS*pA was a functional promoter since it showed similar promoter activity as *PbGDPS*p in the scented *P. bellina* flower tissues (Chuang et al., unpublished). Both results indicated that the down-regulation of the corresponding upstream activators of *GDPS* was responsible for the extremely low *GDPS* expression in *P. aphrodite* (**Figure 5C**).

The possibility of the five bZIPs regulating *GDPS* was evaluated by comparing their gene expression between *P. bellina* and *P. aphrodite* (**Figure 5C**). Among them, *bZIP4*, *bZIP26*, *bZIP29*, and *bZIP32* showed higher expression in *P. bellina* than in *P. aphrodite.* However, both *bZIP29* and *bZIP32* also expressed in floral bud of *P. aphrodite* but still *GDPS* did not express (**Figure 5C**), suggesting that both *bZIP29* and *bZIP32* did not transactivate *PaGDPSpA* promoter. Both PbbZIP29 and PbbZIP32 thus were excluded for further analysis.

### PbbZIP4 Was Able to Distinguish the Promoter Containing the Dual Repeat

**FIGURE 6:**
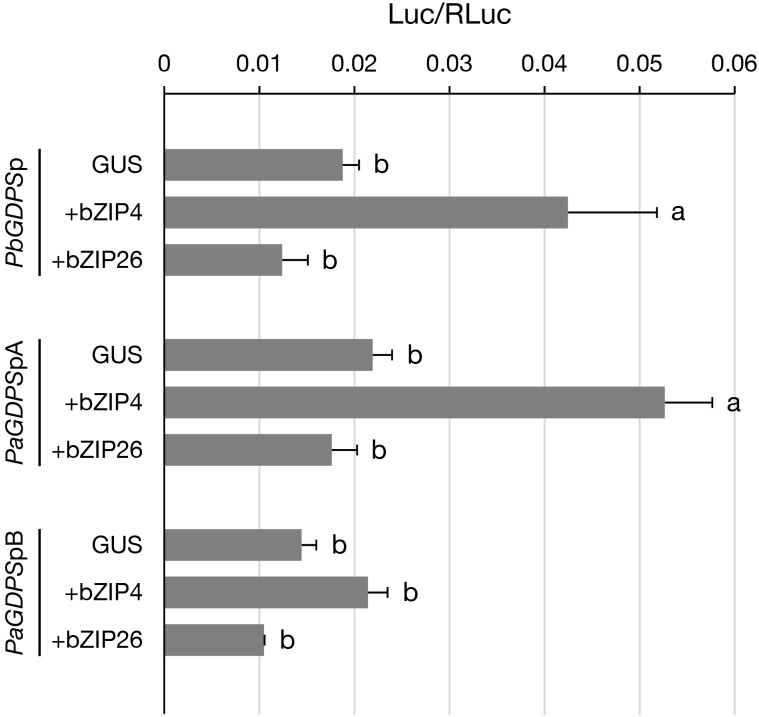
Transient assay for analyzing the transactivation ability of PbbZIP4 and PbbZIP26 on *PbGDPS*p, *PaGDPS*pA, and *PaGDPS*pB. The promoter structure of *PbGDPS*p, *PaGDPS*pA, and *PaGDPS*pB was schematically represented in **Figure 1**. Activation level was given by the ratio of Luc/RLuc. GUS indicated that the assay was performed without the transcription factor. Experiments were based on at least three biological replicates. Statistic tests were performed by using Tukey’s honestly significant difference test at α = 0.05.

Previously, we have shown that PbbZIP4 was able to transactivate *PbGDPS* promoter (Chuang et al., unpublished). Here, to further examine the transactivating ability of PbbZIP4 and PbbZIP26 on various *GDPS* promoters, dual luciferase assay was performed in the floral tissues of the scentless *P. aphrodite*. In the presence of PbbZIP4, it enhanced the promoter activities of both *PbGDPS*p and *PaGDPS*pA, but revealed no effects on *PaGDPS*pB (**Figure 6**). This was consistent with the previous results, in which the activities of both *PbGDPS*p and *PaGDPS*pA were higher than *PaGDPS*pB in the scented *P. bellina* floral tissues (Chuang et al., unpublished). In contrast, PbbZIP26 did not transactivate *PbGDPS*p, *PaGDPS*pA, or *PaGDPS*pB (**Figure 6**). These results indicated that PbbZIP4 was able to distinguish the *GDPS* promoter containing the full dual repeat (*PbGDPS*p), or near full-dual-repeat (*PaGDPS*pA), from the solely one repeat unit (*PaGDPS*pB).

### The Close Association Between the *Trans*-Factor and the *Cis*-Element for Monoterpene Phenotype

The *cis*-element and *trans*-factor for *GDPS* promoter were both identified in the scented *P. bellina*. To investigate how these two factors affected the monoterpene phenotype among the 12 *Phalaenopsis* orchids, we examined the expression levels of *bZIP4* (**Figure 2D**) to establish its correlation with the presence of the dual repeat on *GDPS* promoter (**Figure 2B**) and *GDPS* expression levels (**Figure 2C**) for monoterpene production (**Figure 2A**). Interestingly, *bZIP4* expressed to various extents among these orchids (**Figure 2D**). Strikingly, only the orchid plants concomitantly harbored *bZIP4* expression and the dual repeat on *GDPS* promoter exhibited high *GDPS* expression, and thus produced monoterpenes, including *P.* Meidarland Bellina Age, *P. bellina*, *P. lueddemanniana*, and *P.* I-Hsin Venus (**Figure 2**).

However, orchid species with similar or even higher *bZIP4* expression levels but without the dual repeat showed low *GDPS* expression, and thus did not emit monoterpenes, including *P. javanica*, *P. amboinensis*, *P. mannii*, and *P. cornu-cervi* (**Figure 2**). In contrast, another four species with extremely low expression levels of *bZIP4*, together with their incomplete dual repeat, contributed to their scentless phenotype, including *P. schilleriana*, *P. aphrodite*, *P. equestris* ‘RO-5’, and *P. equestris* ‘WY-7’ (**Figure 2**). Collectively, these results indicated that not only the dual repeat in *GDPS* promoter but also the TFs are crucial for monoterpene production in *Phalaenopsis* orchids.

### Transient Ectopic Expression of *PbbZIP4* in the Scentless *P. aphrodite*

**FIGURE 7:**
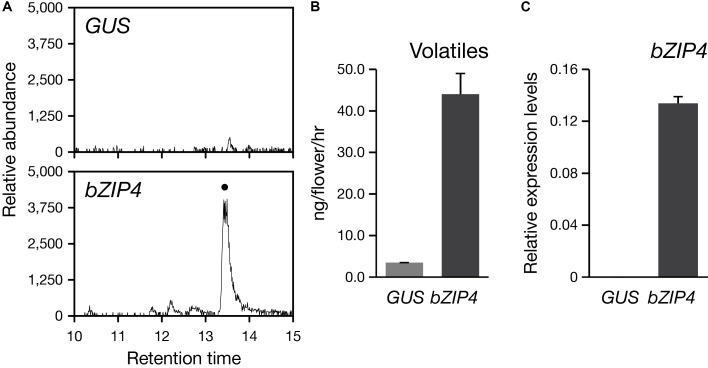
Transient expression of *PbbZIP4* in the scentless *P. aphrodite* flowers induced the production of monoterpeneoids. **(A)** Selected ion (m/z 136) GC-MS profiles of volatiles detected from *GUS*- and *PbZIP4*-expression flowers. The peak with a dot indicates α-terpineol. The products were confirmed by comparing the mass spectra of the indicated peak with the one of the library hit. **(B)** The levels of volatiles emitted from the flowers infiltrated with *GUS* (gray) and *PbbZIP4* (dark-gray). **(C)** Relative expression levels of *PbbZIP4* of the flowers infiltrated with *GUS* (gray) and *PbbZIP4* (dark-gray). Error bars on each column represent ± SE from three biological replicates.

So far, the stable genetic transformation for *Phalaenopsis* orchids is with low efficiency. In addition, *Phalaenopsis* orchids have a long-life cycle with the regeneration time is about 2–3 years, especially if we want to examine the floral phenotype. Instead, the transient ectopic expression system in floral tissues has been successfully established for the study of three MYB TFs regulating the pigmentation patterning in *Phalaenopsis* orchids (Hsu et al., 2015). Thus, to confirm the role of PbbZIP4 *in planta*, we performed a transient assay by infiltrating the Agrobacterium into the flower tissues of the scentless *P. aphrodite.* We analyzed the volatile terpenes emitted from *PbbZIP4*-expressing *P. aphrodite* flowers and detected a 10-fold induction of α-terpineol (a monoterpenoid) as compared to the *GUS* control (**Figures 7A,B**). Furthermore, the raise in the levels of monoterpenes in the infiltrated tissues was indeed resulted from the large increase of *PbbZIP4* transcripts (**Figure 7C**). Therefore, we concluded that PbbZIP4 was involved in the scent production in *Phalaenopsis* orchids.

## Discussion

### The Key Role of the Repeat Unit in the *GDPS* Promoter Activity

In this study, we studied the molecular mechanism determining the scent phenotype in *Phalaenopsis* orchids. We found that a dual repeat of *cis*-element on the *GDPS* promoter played a vital role for floral monoterpene production. The orchids without monoterpene production harbored defective dual repeat and noticeably, most occurring in the R1 region. Serial deletion analysis showed that the removal of R1 unit decreased the promoter activity by 67%, and the further deletion of R2 unit caused an additional 16% reduction, which implies that the R1 unit is crucial for high levels of *GDPS* promoter activity and that the R2 unit is required for the 100% transactivation activity.

### The Evolutionarily Conserved *GDPS* Promoter Sequences Among *Phalaenopsis* Orchids

The *GDPS* promoter fragments isolated from the 10 native *Phalaenopsis* species shared extremely high similarities (90–100% identities, data not shown), which indicates the conservation of the *GDPS* promoter sequences among the *Phalaenopsis* orchids. The 10 native *Phalaenopsis* orchids belong to two-pollinia *Polychilos* and subgenus *Phalaenopsis* (Supplementary Table 4) (Christenson, 2001). According to the evolutionary trend deduced by pollinia number, molecular evidences and biogeography, the four-pollinia basal subgenus *Aphyllae* in South China and Indochina is developed into two-pollina groups during the dispersal into Southeast Asia, including subgenus *Polychilos* in Indonesia and Malaysia, and subgenus *Phalaenopsis* in the Philippines, respectively (Tsai, 2003, 2011; Tsai et al., 2010). As both two-pollina subgenus shared similar *GDPS* promoter sequences, it is possible that this conserved *GDPS* promoter was inherited from their common ancestor of four-pollinia basal group.

### Possible Origin of the Dual Repeats

Repetitive DNA is accounting for a substantial proportion in the whole genomic DNA in most eukaryotes. A number of genetic diseases are related to the large copy number of repetitive sequences, such as Huntington’s disease, fragile X syndrome, and myotonic dystrophy (Pelley, 2007). There are two main classes of repetitive DNA, interspersed repeats dispersed throughout the genome, and tandem repeats located in one area of DNA (also known as satellite DNA) (Bhagavan and Ha, 2011). The interspersed repeats include short interspersed nuclear elements (100- to 500-bp in length) and long interspersed nuclear elements (6000- to 7000-bp in length), and both belong to transposons (Pelley, 2007). On the other hand, satellite DNA is divided into three groups based on the length of the repeat unit, including macrosatellites, minisatellites, and microsatellites. Minisatellites are consisted of repeat sequences ranged from 9 bp to 80 bp (Bhagavan and Ha, 2011), and the size normally ranged from 1-kb to 20-kb. In cauliflower, a Harbinger DNA transposon in the promoter of an R2R3-MYB TF, *purple*, leads to an increase in the gene expression and produces the purple phenotype (Chiu et al., 2010). Two reports have described that a minisatellite-like structure on the promoter of an anthocyanin-regulating TF, *MYB10*, is required for the red color formation in the fruit flesh and leaves in apple and crabapple, respectively (Espley et al., 2009; Tian et al., 2017).

The 150-bp dual repeat identified here consisted of two 75-bp repeat units adjacent to each other, and thus was not considered as a transposon but defined as a minisatellite-like structure. Indeed, this dual repeat did not respond to any known transposon sequences by BLAST against the repetitive sequence database of RepBase (Jurka, 1998; Bao et al., 2015). We speculate that this dual repeat was generated by a tandem duplication event, and have not undergone additional mutation yet for multiple copies.

Six out of 10 *Phalaenopsis* orchids had the dual repeat or near-full dual repeat, including *P. bellina* and *P. luddemanian*, and *P. javanica*, *P. mannii*, *P. aphrodite*, and *P. equestris*, respectively. These six orchids either belong to subgenus *Polychilos* or subgenus *Phalaenopsis* (Supplementary Table 4), suggesting that the dual repeat was retained from their common predecessors, subjected to further mutation, and became defective in the orchids without monoterpene production. As two species of the basal four-pollina subgenus *Aphyllae* (Tsai et al., 2010), *P. hainanensis* and *P. wilsonii*, also emits fragrance (information from http://www.orchid.url.tw/), it will be interesting to study whether they have the dual repeat as well, just similar to the cases in *Capsella* (Brandvain et al., 2013; Sas et al., 2016) and *Petunia* genus (Amrad et al., 2016) describing the loss of the floral scent during the shift of pollinator types.

### Tandem Repeats Correlated to Transcriptional Regulation

In *Phalaenopsis* orchids, the dual repeat on *PbGDPS* promoter is required for its 100% promoter activity. Several studies have also shown that the increasing number of tandem repeats lead to a stepwise increase in promoter activity, and subsequently in gene expression levels. In citrus, three copies of a 20-bp enhancer element on the promoter of lycopene β-cyclases (*CsLCYb1*), an enzyme representing a branch point for carotenoid biosynthesis, are important for its promoter activity (Lu et al., 2016). In *C. roseus*, the copy number of a simple sequence repeats (CT) on the promoter of *Tryptophan decarboxylase*, the first step in the indole alkaloids biosynthesis pathway, is strongly associated its expression levels (Kumar and Bhatia, 2016). Furthermore, the copy number of the *cis*-elements even determines a specific phenotype. For instance, in apple, five direct tandem repeats on the promoter of *MYB10*, an anthocyanin-regulating TF, is only present in the red-fleshed apple varieties, and cause itself autoregulation (Espley et al., 2009). In cotton, two 228-bp tandem repeats on the promoter of an anthocyanin-regulating TF, Red Leaf Cotton 1, is critical for its promoter activity, and only present in the red leaf variety (Gao et al., 2013). Similarly, in our study, the dual repeat is essential for the monoterpene production in *Phalaenopsis* orchids.

### The Dual Repeat Could Be a Novel *Cis*-Acting Sequence

We performed Y1H screening to identify the TFs binding to the dual repeat, and member of bZIP, and MADS-box TFs were isolated. Here, we excluded the possibility of AtAGL81 (MADS-box TF) to interact with *PbGDPS* promoter since it was failed to be isolated from *P. bellina* floral transcriptome in *E*-value cutoff of 1e^-5^. Moreover, we were not able to isolate the similar sequences to AtAGL81 from *P. aphrodite* transcriptome, whose transcript profiles were analyzed in leaf, root, flower bud, and fully open flower (Orchidstra 2.0) (Chao et al., 2017) and *P. equestris* genome (Cai et al., 2015).

The presence of *cis*-elements on the dual repeat was examined by PlantPan analysis (Chow et al., 2015). Several types of TFs were predicted to interact with the dual repeat, including MYB-related, Dof zinc finger protein, GATA, zinc-finger homeodomain protein, squamosa promoter binding protein, and nuclear TF Y subunit beta. Interestingly, we did not find any bZIP-binding *cis*-elements, suggesting that the dual repeat could be a novel *cis*-acting sequence. As the length of the dual repeat is 150-bp, it is possible that other types of TFs may be involved in the scent regulating phenotype, and it awaits further analysis.

### Application for Molecular Marker-Assisted Breeding

The molecular markers to distinguish scent traits have been well developed in rice (Garland et al., 2000; Cordeiro et al., 2002; Jin et al., 2003; Bradbury et al., 2005; Shi et al., 2008; Sakthivel et al., 2009; Myint et al., 2012), and also in other crops, such as soybean (Arikit et al., 2011; Juwattanasomran et al., 2011, 2012), sorghum (Yundaeng et al., 2013), cucumber (Pramnoi et al., 2013; Yundaeng et al., 2015), coconut (Vongvanrungruang et al., 2016), and winter melon (Ruangnam et al., 2017). Just like other modern floriculture cultivars, negative correlation between floral scent and other favorable traits resulted in the difficulty in the scented orchids breeding. The introduction of the scent trait to a well-commercialized cultivar is an alternative approach (Hsiao et al., 2011b). However, the long duration from seedling to first blooming for confirming the floral traits are time consuming and cost inefficient under conventional breeding.

Here, PCR amplification of the dual repeat showed fragment length polymorphism. The two native *Phalaenopsis* orchids and two cultivars emitting monoterpenes showed the 150-bp fragment length of dual repeat, which indicates its potential to be developed as a molecular marker for scent trait. After the link between the dual repeat and the monoterpene emission further verified in more *Phalaenopsis* species and cultivars, the dual repeat could be applied as a molecular marker for early characterization of the scent phenotype in the seedlings and thus benefiting the breeding of scented *Phalaenopsis* orchids.

## Author Contributions

Y-CC, W-CT, W-HC, and H-HC conceived the research plans. Y-CC performed most of the experiment, analyzed the data, and wrote the article with contributions of all the authors. Y-CH and C-YH assisted in the identification of the dual repeat and the analysis in the gene expression and scent compounds in *Phalaenopsis* orchids. C-MY, NM, and MO-T performed the yeast one hybrid screening analysis and provided valuable comments on the manuscript. H-HC supervised and complemented the writing. All authors read and approved the submitted version.

## Conflict of Interest Statement

The authors declare that the research was conducted in the absence of any commercial or financial relationships that could be construed as a potential conflict of interest.

## Footnotes

1 http://www.orchideya.ca/

2 http://www.ebi.ac.uk/Tools/msa/clustalo/

3 https://embnet.vital-it.ch/software/BOX_form.html

## Abbreviations

bZIP: basic leucine zipper
DMADP: dimethylallyl diphosphate
GDPS: geranyl diphosphate synthase
IDP: isopentenyl diphosphate
*P*.,: *Phalaenopsis*
TFs: transcription factors
Y1H: yeast one-hybrid
