# Supplementary material for: A Dual Repeat Cis-Element Determines Expression of GERANYL DIPHOSPHATE SYNTHASE for Monoterpene Production in Phalaenopsis Orchids
Source: Front Plant Sci. 2018 Jun 5;9:765. doi: 10.3389/fpls.2018.00765 (PMC5996158; doi:10.3389/fpls.2018.00765)
Supplement: Supplementary file 1 [file Data_Sheet_1.pdf]

Supplementary Material

A dual repeat *cis*-element determines expression of *GERANYL DIPHOSPHATE SYNTHASE* for monoterpene production in *Phalaenopsis* orchids

Yu-Chen Chuang, Yi-Chu Hung, Chi-Yu Hsu, Chuan-Ming Yeh, Nobutaka Mitsuda, Masaru Ohme-Takagi, Wen-Chieh Tsai, Wen-Huei Chen, and Hong-Hwa Chen*

*** Correspondence:** Dr. Hong-Hwa Chen, hhchen@mail.ncku.edu.tw

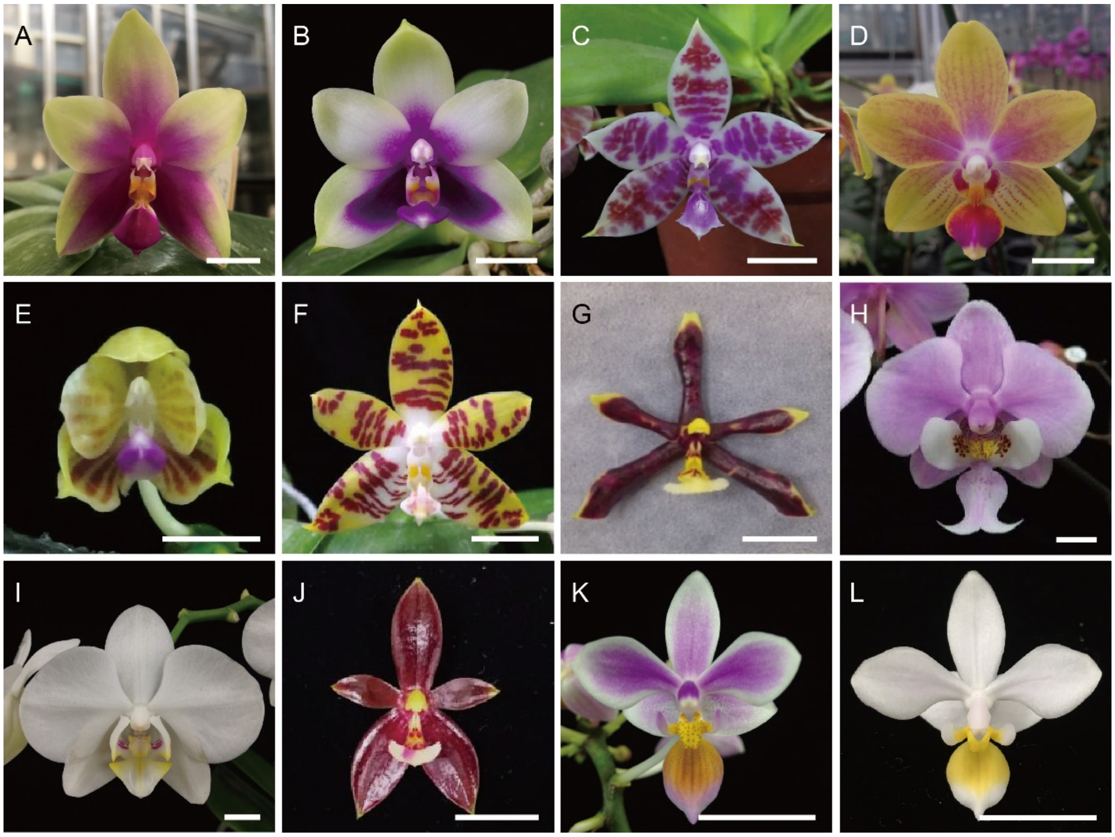

Supplementary Figure 1. The 12 *Phalaenopsis* orchids used in this study. The order of the figures followed the presentation in Fig. 2. **(A)** *P.* Meidarland Bellina Age 'LM128', **(B)** *P. bellina*, **(C)** *P. lueddemanniana*, **(D)** *P.* I-Hsin Venus, **(E)** *P. javanica*, **(F)** *P. amboinensis* var. *yellow*, **(G)** *P. mannii*, **(H)** *P. schilleriana*, **(I)** *P. aphrodite* subsp. *formosana*, **(J)** *P. cornu-cervi* var. *red*, **(K)** *P. equestris* ‘RO-5’, and **(L)** *P. equestris* ‘WY-7’. Scale bar = 1 cm.

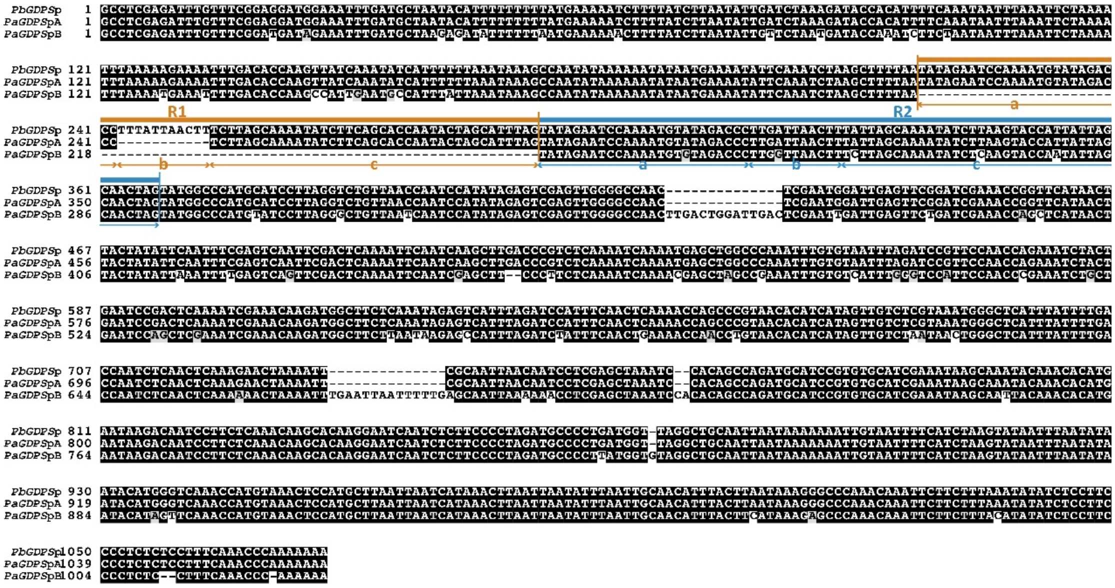

Supplementary Figure 2. The sequence alignment of *PbGDPS*p, *PaGDPS*pA and *PaGDPS*pB. The two units of the dual repeat are labeled with the thick color bars above the alignment, which orange indicates R1, and blue indicates R2. The subunits of R1 and R2 are labeled with the color lines under the alignment. The sequence alignment was generated by using Clustal Omega and displayed by using BOXSHADE.

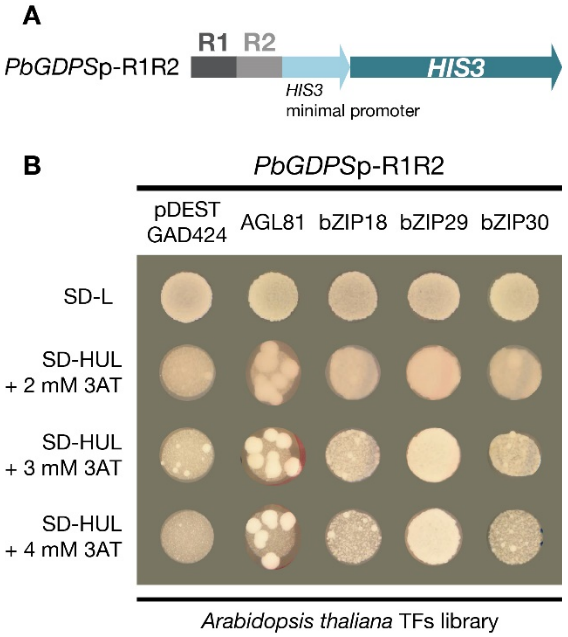

Supplementary Figure 3. The identification of TFs binding to the dual repeat of *PbGDPS* by Y1H screening. **(A)** The dual repeat of *PbGDPS* was used as a bait for isolating TFs binding to it. **(B)** The TFs binding to the dual repeat region of *PbGDPS* promoter were isolated by yeast one-hybrid screening using *Arabidopsis* TF-only library. SD-HUL indicated SD medium without histidine, uracil and leucine.

Supplementary Table 1 Sources of the 12 *Phalaenopsis* orchids used in this study.

	**Name** (by alphabetical order)	**Source** (Taiwan)
Species	*P. amboinensis* var. *yellow*	Tung-Hai Orchids
	P. aphrodite subsp. *formosana*	Taiwan Sugar Corp.
	P. bellina	Ming-Hui Orchids Nursery
	P. cornu-cervi var. *red*	Mi-Tuo Orchids
	P. equestris ‘RO-5’	Taiwan Sugar Corp.
	P. equestris ‘WY-7’	Taiwan Sugar Corp.
	P. javanica	Mi-Tuo Orchids
	P. lueddemanniana	Mi-Tuo Orchids
	P. mannii	Ji An Guang Feng
	P. schilleriana	Han-Lin Orchids
Hybrid	*P.* I-Hsin Venus	I-Hsin Biotechnology Corp.
	P. Meidarland Bellina Age 'LM128'	Meidarland Orchids

Supplementary Table 2 List of oligonucleotide primers used in this study.

**Purpose**	Sequence (5' to 3')	
Target	Forward	Reverse
**Detection of *GDPS* gene, promoter and dual-repeat**		
Gene	ATGGCAGCAATCTTTCCCTCAATCCCCTCCAATTT	CGAGGGGAGGGGCGGTGCG
Promoter	GCCTCGAGATTTGTTTCGG	CCATGGTTTTTTTGGGTTTGAAAGGAGAG
Dual repeat	TTGCCTCGAGATTTGTTTCGGAGGATGGA	ACCTAAGGATGCATGGGCCATACTAG
**Real-time PCR**		
Actin1	CCTCAAATCTCCCAAACCCTAA	CGATGCGGAGAGATAGGATTG
GDPS	GCTGAGGGAGGCAAGGATAGAT	GCACCCAGCAGCATGAAGATC
bZIP4	CACGCAGTTTTCCAACGGTAAAG	AACTCCCACCATGATTGGGAAGC
**Transient assay construction**		
*PbG*p-2010	GGATCCGACACATGAAAATCATGTTTGAT	CCATGGTTTTTTTGGGTTTGAAAGGAGAG
*PbG*p-1076	GGATCCGCCTCGAGATTTGTTTCGG	CCATGGTTTTTTTGGGTTTGAAAGGAGAG
*PbG*p-859	GGATCCTATAGAATCCAAAATGTATAGACCCT	CCATGGTTTTTTTGGGTTTGAAAGGAGAG
*PbG*p-836	GGATCCCTTTATTAACTTTCTTAGCAAAATATCTTCAGC	CCATGGTTTTTTTGGGTTTGAAAGGAGAG
*PbG*p-822	GGATCCCTTAGCAAAATATCTTCAGCA	CCATGGTTTTTTTGGGTTTGAAAGGAGAG
*PbG*p-784	GGATCCGAATCCAAAATGTATAGACCCTTG	CCATGGTTTTTTTGGGTTTGAAAGGAGAG
*PbG*p-760	GGATCCCTTGATTAACTTTATTAGCAAAATATCTTAA	CCATGGTTTTTTTGGGTTTGAAAGGAGAG
*PbG*p-747	GGATCCATTAGCAAAATATCTTAAGTACCATTATTAG	CCATGGTTTTTTTGGGTTTGAAAGGAGAG
*PbG*p-729	GGATCCGTACCATTATTAGCAACTAGTATGGC	CCATGGTTTTTTTGGGTTTGAAAGGAGAG
*PbG*p-710	GGATCCGCCCATGCATCCTTAGGTCTGTTAA	CCATGGTTTTTTTGGGTTTGAAAGGAGAG
*PbG*p-584	GGATCCGACTCAAAATTCAATCAAGCTT	CCATGGTTTTTTTGGGTTTGAAAGGAGAG
*PbG*p-410	GGATCCCACATCATAGTTGTCTCGTAAATGGGCTC	CCATGGTTTTTTTGGGTTTGAAAGGAGAG
*PbG*p-354	GGATCCGAACTAAAATTCGCAATTAACAATCCCCG	CCATGGTTTTTTTGGGTTTGAAAGGAGAG
*PbG*p-297	GGATCCGTGCATCGAAATAAGCAAATAC	CCATGGTTTTTTTGGGTTTGAAAGGAGAG
*PbG*p-216	GGATCCGATGCCCCTGATGGTTAGGCTGC	CCATGGTTTTTTTGGGTTTGAAAGGAGAG
**Yeast one hybrid**		
Dual repeat	CCCGGGTATAGAATCCAAAATGTATAGACC	TCTAGACTAGTTGCTAATAATGGTACTTAA
**Transactivation assay of PbbZIP4 and PbbZIP26**		
*PbGDPS*p	GGATCCGCCTCGAGATTTGTTTCGG	CCATGGTTTTTTTGGGTTTGAAAGGAGAG
*PaGDPS*pA	GGATCCGCCTCGAGATTTGTTTCGG	CCATGGTTTTTTTGGGTTTGAAAGGAGAG
*PaGDPS*pB	GGATCCGCCTCGAGATTTGTTTCGG	CCATGGTTTTTTTGGGTTTGAAAGGAGAG
PbbZIP4	TCTAGAATGGACGCGAATCGGCCGA	CCCGGGTCACATAAAACTCCCACCATG
PbbZIP26	TCTAGAATGCAGACGAATTCCATAGATCCATC	TCTAGATCAGAAGTTGCTGCTGCTCTCA

Supplementary Table 3 Floral volatiles of 12 *Phalaenopsis* orchids.

Species / ng/flower/hr	P.Meidarland Bellina Age	*P. belllina*	*P. lueddema-nniana*	P. I-Hsin Venus	*P. javanica*	*P. amboinensis*	*P. mannii*	*P. schilleriana*	*P. aphrodite*	*P. cornu-cervi*	*P. equestris* 'RO-5'	*P. equestris* 'WY-7'
**Monoterpenoids**												
Eucalyptol	—[g]	—	—	65.77	—	—	—	—	—	—	—	—
Geraniol[a]	556.97	2591.41	723.04	—	—	—	—	—	—	—	—	—
Limonene	—	16.77	—	—	—	—	—	—	—	—	—	—
Linalool[b]	3003.40	946.55	—	559.59	—	—	—	—	—	—	—	—
Myrcene	—	321.48	—	—	—	—	—	—	—	—	—	—
Neral	101.19	21.30	12.31	—	—	—	—	—	—	—	—	—
Ocimene[c]	—	231.47	—	—	—	—	—	—	—	—	—	—
α-Terpineol	68.33	—	—	—	—	—	—	—	—	—	—	—
**Sesquiterpenoids**												
α-Amorphene	—	—	—	—	45.29	—	—	—	—	—	—	—
γ-Cadinene	—	—	—	—	—	21.24	—	—	—	—	—	—
Farnesene[d]	284.01	—	—	—	—	—	—	—	—	—	—	—
Muurolene[e]	—	—	—	—	—	19.72	—	—	—	—	—	—
**Benzenoids**												
Benzaldehyde	179.42	—	—	66.35	15.78	36.19	6.09	8.57	—	—	—	—
**Phenylpropanoid**												
Cinnamaldehyde[f]	78.23	—	—	—	—	—	68.00	—	—	—	—	—
**Fatty acid derivates**												
methyl-Myristate	—	—	—	—	—	—	76.19	—	—	—	—	—

a: Include both geraniol and granial.

b: Include linalool and linalool oxide.

c: Include (*E*)-β-ocimene, (*Z*)-β-ocimene, and allo-ocimene.

d: Include (*E*,*E*)-α-farnesene, farnesol, farnesal, and hexa-hydro-farnesol.

e: Include T-muurolol and γ-muurolene

f: Include (*E*)-cinnamaldehyde and cinnamyl acetate

g: This compound was not identified.

Supplementary Table 4 The classification of the 10 native *Phalaenopsis* species used in this study.

Taxa and systematic classification[a]	Geographical distribution[b]
**Subgenus *Polychilos***	
**Section *Polychilos***	
P. cornu-cervi	Northeast India and the Nicobar Islands to Java and Borneo
P. mannii	Northeast India, Nepal and China to Vietnam
**Section *Amboinenses***	
P. amboinensis	Indonesia
P. bellina	Malaysia
P. javanica	Endemic to Indonesia (Java)
P. lueddemanniana	Endemic to Philippines
**Subgenus *Phalaenopsis***	
**Section *Phalaenopsis***	
P. aphrodite	Southeastern Taiwan
P. schilleriana	Endemic to Philippines
**Section *Esmeralda***	
P. equestris	Southeastern Taiwan

a: This classifications is based on Christenson (2001).

b: This geographical distribution is based on Tsai (2011).

This table is revised from Molecular phylogeny and biogeography of *Phalaenopsis* species. Tsai, C. C. Chen, W. H. and Chen H. H., eds. Copyright @ 2011 Singapore, World Scientific.
